# Supplementary material for: Tissue-specific profiling of age-dependent miRNAomic changes in Caenorhabditis elegans
Source: Nat Commun. 2024 Feb 1;15:955. doi: 10.1038/s41467-024-45249-4 (PMC10834975; doi:10.1038/s41467-024-45249-4)
Supplement: Supplementary file 1 — Supplementary Information [file 41467_2024_45249_MOESM1_ESM.pdf]

Supplementary Information

Tissue-specific profiling of age-dependent miRNAomic changes in *Caenorhabditis elegans*

Xueqing Wang[1,2†], Quanlong Jiang[3,4†], Hongdao Zhang[1,2†], Zhidong He[1,2], Yuanyuan Song[1,2], Yifan Chen[1,2], Na Tang[1,2], Yifei Zhou[1,2], Yiping Li[1,2], Adam Antebi[5,6], Ligang Wu[1,2]*, Jing-Dong J. Han[4]*, Yidong Shen[1,2]*

Affiliations:

[1] State Key Laboratory of Cell Biology, Shanghai Institute of Biochemistry and Cell Biology, Center for Excellence in Molecular Cell Science, Chinese Academy of Sciences

200031 Shanghai, China

[2] University of Chinese Academy of Sciences

100049 Beijing, China

[3] CAS Key Laboratory of Computational Biology, Shanghai Institute of Nutrition and Health, Shanghai Institutes for Biological Sciences, Chinese Academy of Sciences

200031 Shanghai, China

[4] Peking-Tsinghua Center for Life Sciences, Academy for Advanced Interdisciplinary Studies, Center for Quantitative Biology (CQB), Peking University

102213 Beijing, China

[5] Max Planck Institute for Biology of Ageing

D-50931 Cologne, Germany

[6] Cologne Excellence Cluster on Cellular Stress Responses in Aging-Associated Diseases (CECAD), University of Cologne

50674 Cologne, Germany.

†These authors contributed equally: Xueqing Wang, Quanlong Jiang, and Hongdao Zhang

*Correspondence to: yidong.shen@sibcb.ac.cn, jackie.han@pku.edu.cn, lgwu@sibcb.ac.cn.

The **Supplementary Information** contains 8 figures, 9 spreadsheets, and a reporting summary.

Supplementary Figures

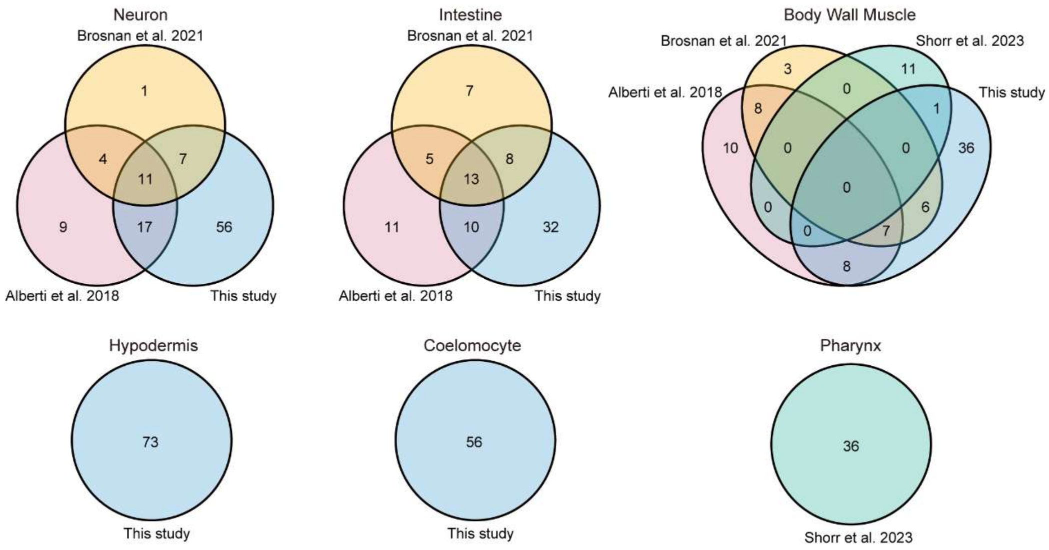

Supplementary Fig. 1. Detected *C. elegans* miRNA genes in three published tissue-specific miRNAomic analysis and this study.

Venn diagrams showing the detected miRNA genes in the indicated studies. Only miRNA genes curated in MirGeneDB 2.1 are counted.

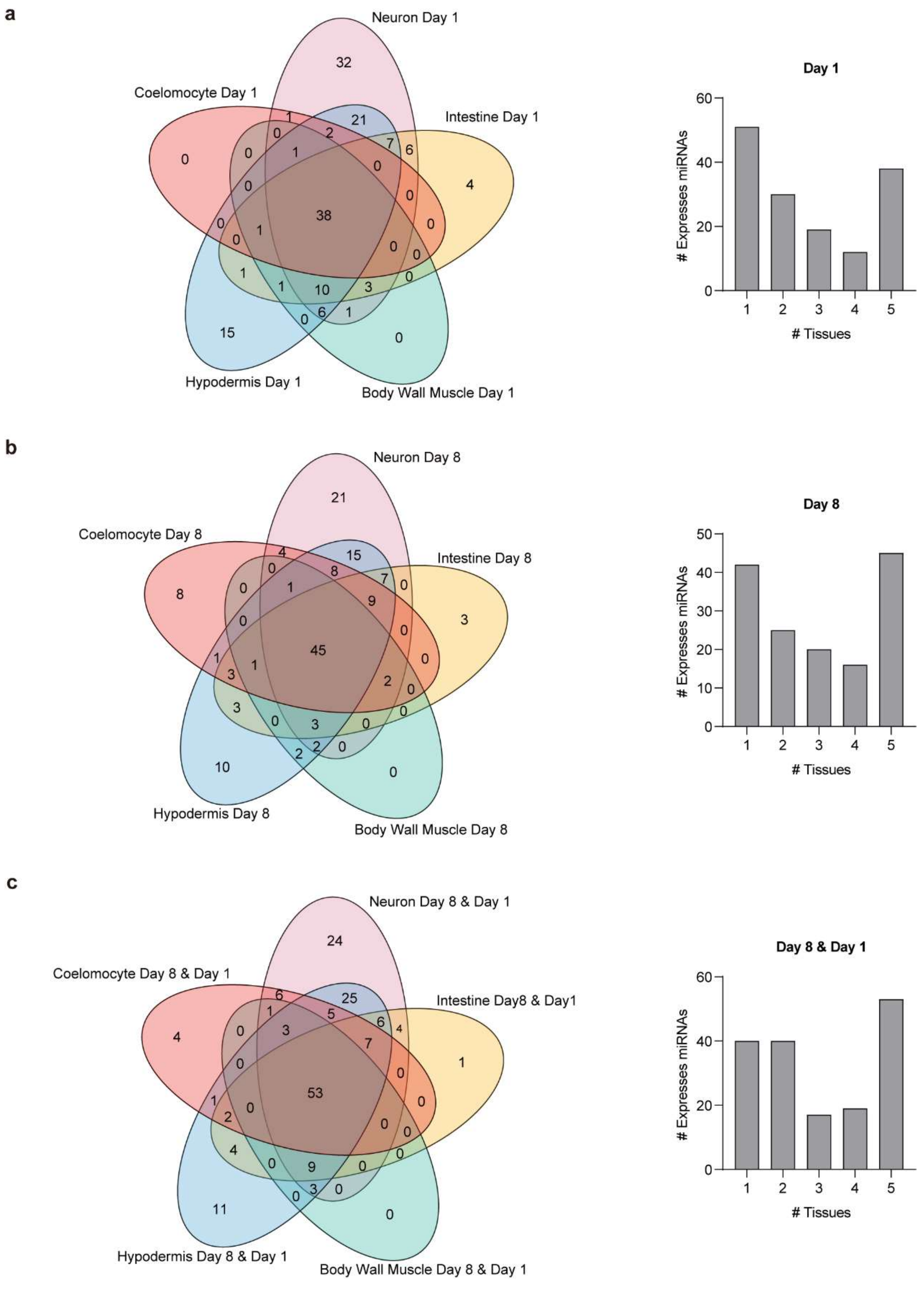

Supplementary Fig. 2. The overlap of detected miRNAs across tissues.

The expressed miRNAs in young (a), aged (b), or either young or aged (c) worm

tissues highly overlap. The numbers of expressed miRNAs shared in 1, 2, 3, 4, or 5 tissues are depicted on the right of each panel.

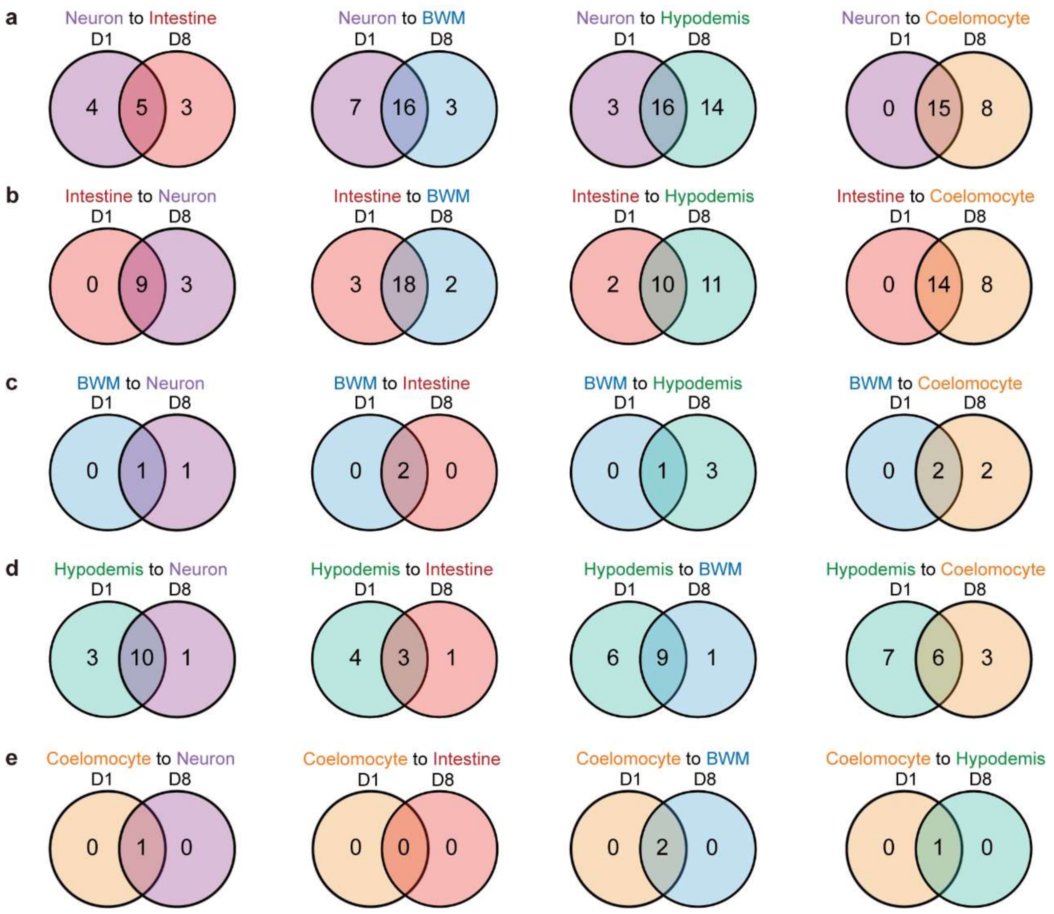

Supplementary Fig. 3. Ageing changes the composition of inter-tissue transported miRNAs.

Venn diagrams comparing the predicted PITT-miRNAs transported from neuron (a), intestine (b), body wall muscle (BWM) (c), hypodermis (d), and coelomocyte (e) to other tissues at day 1 (D1) and day 8 (D8) of adulthood.

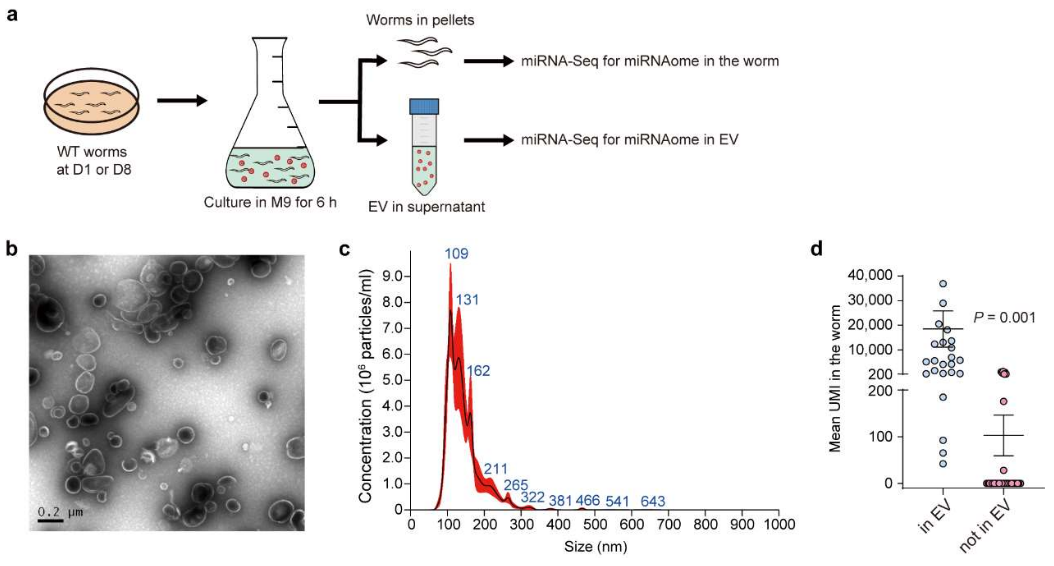

Supplementary Fig. 4. The purification of extracellular vesicles from *C. elegans*.

a. A flowchart depicting the preparation of worm and EV samples for miRNA-Seq. D1: day 1 of adulthood, D8: day 8 of adulthood. Under the Creative Commons Attribution 4.0 International License (http://creativecommons.org/licenses/by/4.0/), some of the elements in the depiction are adapted from Zhou et al., 2019, with minor modifications.

b. A typical transmission electron microscope image of the isolated worm EVs. Note that worm EVs are with a typical cup shape. Scale bar: 0.2 μm.

c. Particle size distribution of isolated EVs from worms.

d. The levels of indicated groups of PITT-miRs in the miRNA-Seq of whole worms. Data from both young and aged worms are used to calculate mean UMI. Note that PITT-miRs undetected in EV showed a much lower expression in the whole worm. The miRNAs with a UMI sum below 10 are considered as not detected and excluded

from other analyses but presented here to show the different expression levels of the two types of PITT-miRs. Error bar: SEM. Unpaired *t*-test. Source data are provided as a Source Data file.

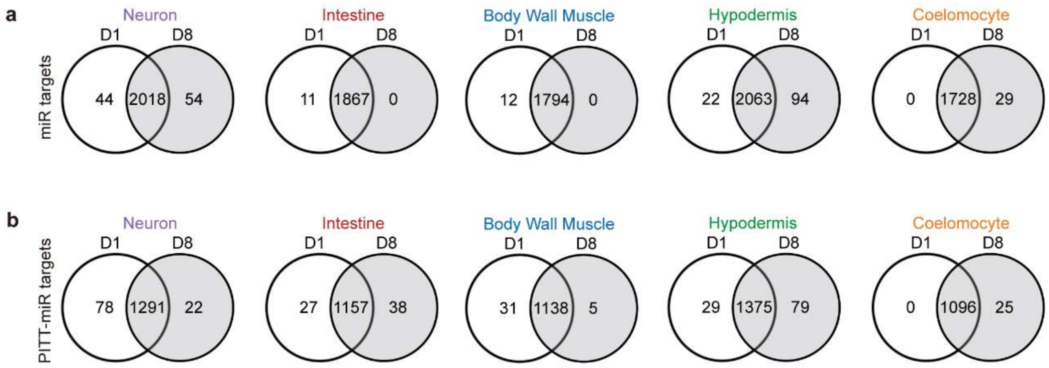

Supplementary Fig. 5. MiRNA targets in young and aged worms are largely overlapping.

Venn diagrams comparing the targets of all miRNAs (a) and PITT-miRs (b) in the indicated worm tissues at day 1 (D1) and day 8 (D8) of adulthood.

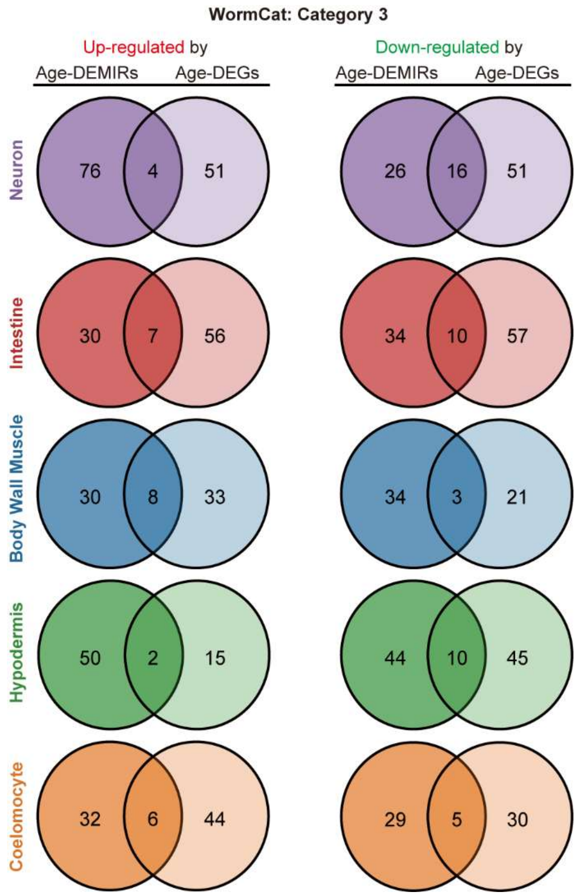

Supplementary Fig. 6. A comparison between the biological processes controlled by Age-DEMIR targets and those by ageing-controlled mRNAs.

Venn diagrams showing the overlapping of the biological processes upregulated (left) or downregulated (right) by Age-DEMIR targets (this study) and those by ageing-controlled mRNAs (Age-DEGs reported in Wang et al., 2022). The biological processes are Category 3 by WormCat.

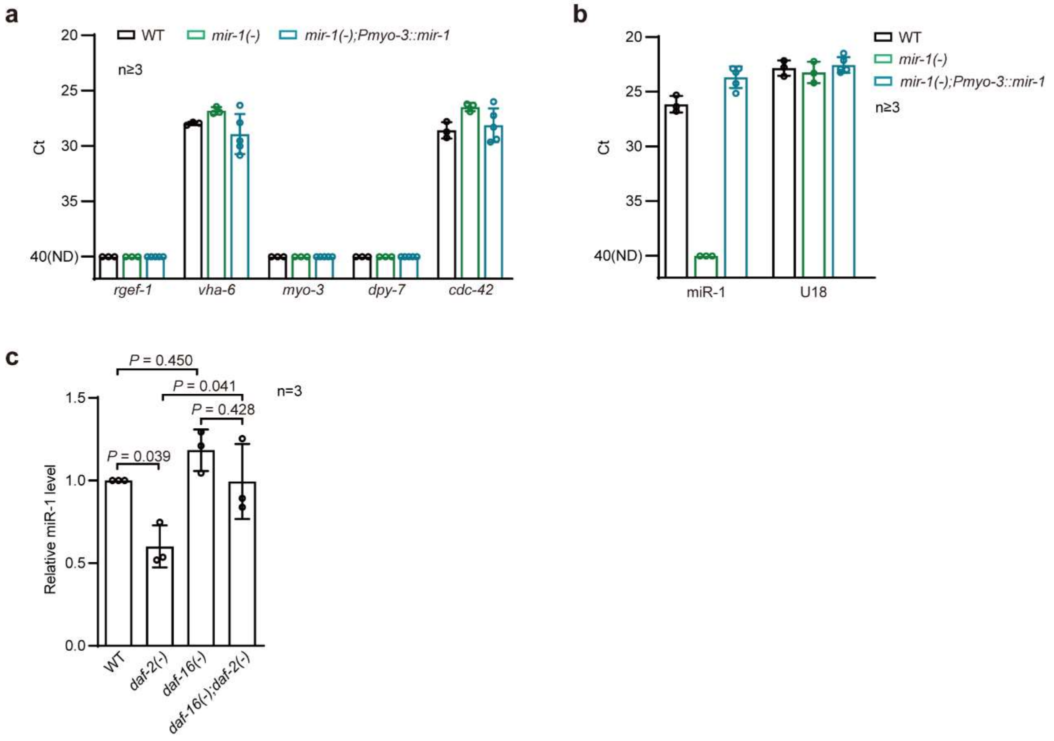

Supplementary Fig. 7. miR-1 could be a messenger of insulin/IGF-1 signalling from BWM to the intestine.

a. The purity validation of isolated intestinal cells from the indicated strains. *rgef-1*, *vha-6*, *myo-3*, and *dpy-7* were respectively used as markers for neurons, intestine, BWM, and hypodermis. *cdc-42* is a housekeeper gene expressed in all tissues. Note that Ct value is reversibly correlated with gene expression level. Ct values of samples without RT-qPCR signal were set at 40.

b. The level of miR-1 in the isolated intestinal cells of the indicated strains.

c. *daf-16* suppresses miR-1 in the longevity mutant of *daf-2(-)*. One-way ANOVA test, correct for multiple comparison with Tukey test.

Source data are provided as a Source Data file.

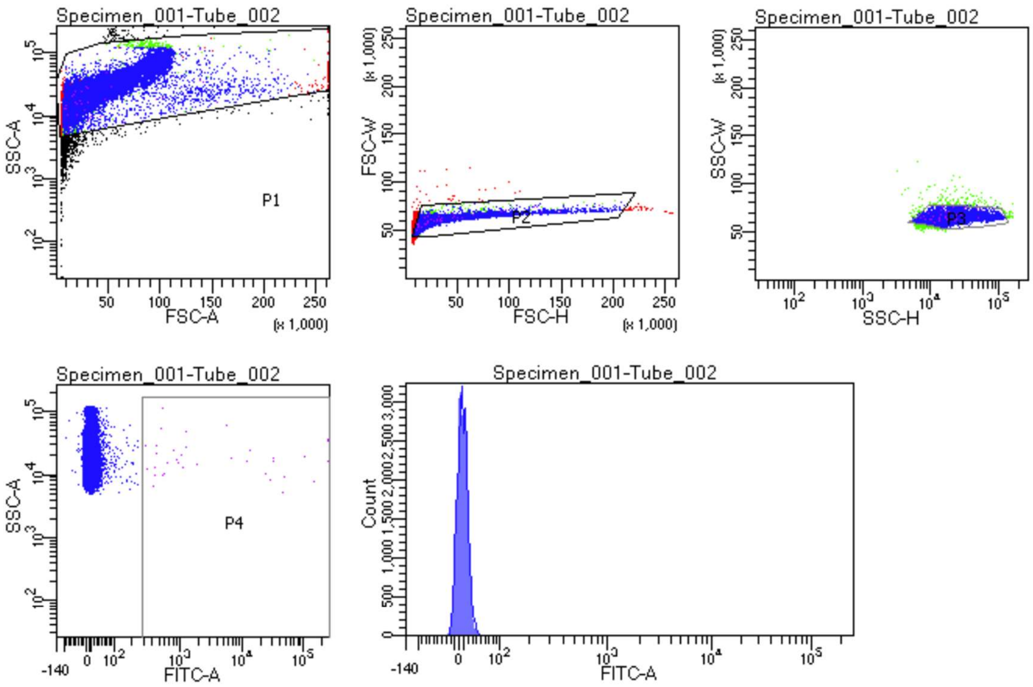

Tube: Tube_002

Population	#Events	%Parent	%Total
All Events	30,314	####	100.0
P1	28,885	95.3	95.3
P2	23,980	83.0	79.1
P3	23,083	96.3	76.1
P4	35	0.2	0.1

Supplementary Fig. 8. A representative gating strategy for FACS sorting of YFP-labelled neurons.

To set the boundaries for gating, N2 worms are as the negative control of auto-fluorescence. First, cell population is identified by SSC-A versus FSC-A plots (P1). Next, doublets are eliminated using FSC-H versus FSC-W and SSC-H versus SSC-W plots (P2 and 3). Then, neurons are gated for YFP signal (FITC-A, P4) and defined for sorting.
