# Supplementary material for: Tissue-specific profiling of age-dependent miRNAomic changes in Caenorhabditis elegans
Source: Nat Commun. 2024 Feb 1;15:955. doi: 10.1038/s41467-024-45249-4 (PMC10834975; doi:10.1038/s41467-024-45249-4)
Supplement: Supplementary file 3 — Description of Additional Supplementary Files [file 41467_2024_45249_MOESM3_ESM.pdf]

Description of Additional Supplementary Files

File name: Supplementary Data 1

Description: The Age-DEMIRs in examined worm tissues.

File name: Supplementary Data 2

Description: A comparison with previous miRNAomic studies in worm tissues.

File name: Supplementary Data 3

Description: The age-dependent change of miRNAomes in EV and worm.

File name: Supplementary Data 4

Description: MiRNA targets in analysed worm tissues.

File name: Supplementary Data 5

Description: Age-DEMIR targets in analysed worm tissues.

File name: Supplementary Data 6

Description: WormCat analysis of Age-DEMIR targets in analysed tissues.

File name: Supplementary Data 7

Description: Other statistics.

File name: Supplementary Data 8

Description: List of worm strains and RT-qPCR primers.

File name: Supplementary Data 9

Description: Additional information on transcriptomic analysis of isolated cells from worm tissues.
