# Supplementary material for: Tissue-specific profiling of age-dependent miRNAomic changes in Caenorhabditis elegans
Source: Nat Commun. 2024 Feb 1;15:955. doi: 10.1038/s41467-024-45249-4 (PMC10834975; doi:10.1038/s41467-024-45249-4)
Supplement: Supplementary file 13 — Reporting Summary [file 41467_2024_45249_MOESM13_ESM.pdf]

nature portfolio

Corresponding author(s): Yidong Shen

Last updated by author(s): Dec 20, 2023

Reporting Summary

Nature Portfolio wishes to improve the reproducibility of the work that we publish. This form provides structure for consistency and transparency in reporting. For further information on Nature Portfolio policies, see our Editorial Policies and the Editorial Policy Checklist.

Statistics

For all statistical analyses, confirm that the following items are present in the figure legend, table legend, main text, or Methods section.

n/a	Confirmed	
☐	☒	The exact sample size (*n*) for each experimental group/condition, given as a discrete number and unit of measurement
☐	☒	A statement on whether measurements were taken from distinct samples or whether the same sample was measured repeatedly
☐	☒	The statistical test(s) used AND whether they are one- or two-sided<br>*Only common tests should be described solely by name; describe more complex techniques in the Methods section.*
☐	☒	A description of all covariates tested
☐	☒	A description of any assumptions or corrections, such as tests of normality and adjustment for multiple comparisons
☐	☒	A full description of the statistical parameters including central tendency (e.g. means) or other basic estimates (e.g. regression coefficient) AND variation (e.g. standard deviation) or associated estimates of uncertainty (e.g. confidence intervals)
☐	☒	For null hypothesis testing, the test statistic (e.g. *F*, *t*, *r*) with confidence intervals, effect sizes, degrees of freedom and *P* value noted<br>*Give P values as exact values whenever suitable.*
☒	☐	For Bayesian analysis, information on the choice of priors and Markov chain Monte Carlo settings
☒	☐	For hierarchical and complex designs, identification of the appropriate level for tests and full reporting of outcomes
☐	☒	Estimates of effect sizes (e.g. Cohen's *d*, Pearson's *r*), indicating how they were calculated

Our web collection on statistics for biologists contains articles on many of the points above.

Software and code

Policy information about availability of computer code

Data collection	No software was used.
Data analysis	For miRNA-Seq on isolated worm tissues, reads were mapped to WBcel235 by STAR (v2.6.0c).<br>For miRNA-Seq of whole worm and EV, reads were mapped to WBcel235 by miRDeep2.<br>Differentially expressed miRNAs were identified using DEGseq.<br>MiRNA targets were identified by TargetScanWorm (Release 6.2).<br>Pathway analysis was performed using WormCat 2.0.<br>ImageJ (1.53t) was used for microscopic image analysis.<br>GraphPad Prism 6.01 (GraphPad Software) was used for all statistic analysis.

For manuscripts utilizing custom algorithms or software that are central to the research but not yet described in published literature, software must be made available to editors and reviewers. We strongly encourage code deposition in a community repository (e.g. GitHub). See the Nature Portfolio guidelines for submitting code & software for further information.

Data

Policy information about availability of data

All manuscripts must include a data availability statement. This statement should provide the following information, where applicable:

- Accession codes, unique identifiers, or web links for publicly available datasets
- A description of any restrictions on data availability
- For clinical datasets or third party data, please ensure that the statement adheres to our policy

The miRNA-Seq data from this publication have been deposited to the SRA database (https://www.ncbi.nlm.nih.gov/sra) and assigned the links as below:
Small RNA-Seq of isolated tissues from worms at D1 and D8 of adulthood:
https://www.ncbi.nlm.nih.gov/sra/?term=PRJNA854735
Small RNA-Seq of extracellular vesicles and worms at D1 and D8 of adulthood:
https://www.ncbi.nlm.nih.gov/sra/?term=PRJNA868152
All other data are available from the corresponding author upon reasonable request.

Research involving human participants, their data, or biological material

Policy information about studies with human participants or human data. See also policy information about sex, gender (identity/presentation), and sexual orientation and race, ethnicity and racism.

Reporting on sex and gender	NA in this study.
Reporting on race, ethnicity, or other socially relevant groupings	NA in this study.
Population characteristics	NA in this study.
Recruitment	NA in this study.
Ethics oversight	NA in this study.

Note that full information on the approval of the study protocol must also be provided in the manuscript.

Field-specific reporting

Please select the one below that is the best fit for your research. If you are not sure, read the appropriate sections before making your selection.

☒ Life sciences ☐ Behavioural & social sciences ☐ Ecological, evolutionary & environmental sciences

For a reference copy of the document with all sections, see nature.com/documents/nr-reporting-summary-flat.pdf

Life sciences study design

All studies must disclose on these points even when the disclosure is negative.

Sample size	The sample size in the current study was determined by referring to well-accepted previous studies.
Data exclusions	No data were excluded.
Replication	All attempts at replication were successful. All experiments were repeated independently for at least three times.
Randomization	All samples/worms were allocated into experimental groups randomly.
Blinding	All the assays in this study were blinded except for all the RNA-Seq assays. For RNA-Seq assays, large amounts of samples were collected and data were analysed by computational algorithms, making blinding not relevant to the study.

Reporting for specific materials, systems and methods

We require information from authors about some types of materials, experimental systems and methods used in many studies. Here, indicate whether each material, system or method listed is relevant to your study. If you are not sure if a list item applies to your research, read the appropriate section before selecting a response.

Materials & experimental systems

n/a	Involved in the study	
☐	☒	Antibodies
☐	☒	Eukaryotic cell lines
☒	☐	Palaeontology and archaeology
☐	☒	Animals and other organisms
☒	☐	Clinical data
☒	☐	Dual use research of concern
☒	☐	Plants

Methods

n/a	Involved in the study	
☒	☐	ChIP-seq
☐	☒	Flow cytometry
☒	☐	MRI-based neuroimaging

Antibodies

Antibodies used	Monoclonal Anti-α-Tubulin Antibody Produced in Mouse, Sigma-Aldich, Cat# T5168;<br>Anti-GFP (B-2), SANTA CRUZ BIOTECHNOLOGY, INC, Cat# sc-9996;<br>Goat anti-Mouse IgG (H+L) Cross-Adsorbed Secondary Antibody, HRP, ThermoFisher Scientific, Cat# G-21040.
Validation	All antibodies were used for western blotting in current study.<br>Anti-α-Tubulin(Mouse Monoclonal), manufacturer's description:https://www.sigmaaldrich.com/catalog/product/sigma/t5168?lang=zh&region=CN<br>Anti-GFP(B-2), manufacturer's description:https://www.scbt.com/scbt/zh/product/gfp-antibody-b-2<br>Goat anti-Mouse IgG (H+L) Cross-Adsorbed Secondary Antibody, HRP, manufacturer's description: https://www.thermofisher.cn/cn/zh/antibody/product/Goat-anti-Mouse-IgG-H-L-Cross-Adsorbed-Secondary-Antibody-Polyclonal/G-21040

Eukaryotic cell lines

Policy information about cell lines and Sex and Gender in Research

Cell line source(s)	HEK293T was obtained from American Type Culture Collection(ATCC).
Authentication	The HEK293T used in the current study was authenticated by ATCC and confirmed prior to experiments by cell morphology.
Mycoplasma contamination	All cells were tested negative for mycoplasma contamination.
Commonly misidentified lines (See ICLAC register)	No commonly misidentified cell lines were used.

Animals and other research organisms

Policy information about studies involving animals; ARRIVE guidelines recommended for reporting animal research, and Sex and Gender in Research

Laboratory animals	Wild type, mutants and trangenic strains of Caenorhabditis elegans were used in the study. All used strains were listed in Supplementary Data 8. The worms used in this study are all hermaphrodites, aged from day 1 to 8 of adulthood.
Wild animals	No wild animals.
Reporting on sex	NA in this study.
Field-collected samples	No field-collected samples.
Ethics oversight	No ethics oversight.

Note that full information on the approval of the study protocol must also be provided in the manuscript.

Flow Cytometry

Plots

Confirm that:

☒ The axis labels state the marker and fluorochrome used (e.g. CD4-FITC).

☒ The axis scales are clearly visible. Include numbers along axes only for bottom left plot of group (a 'group' is an analysis of identical markers).

☒ All plots are contour plots with outliers or pseudocolor plots.

☒ A numerical value for number of cells or percentage (with statistics) is provided.

Methodology

Sample preparation	Worms with YFP-labelled neurons were incubated with SDS-DTT and proteolysis with mechanical disruption. Worm lysates were filtered with a 5-µm cell strainer. Filtered worm lysates were subjected to FACS.
Instrument	FACS Aria III
Software	BD FACSDiva 8.0.1
Cell population abundance	Sorted neurons were of 0.1%~0.2% of parent events.
Gating strategy	Please see Supplementary Fig. 8, which exemplifies the gating strategy.

☒ Tick this box to confirm that a figure exemplifying the gating strategy is provided in the Supplementary Information.
